# Comparing the intestinal transcriptome of Meishan and Large White piglets during late fetal development reveals genes involved in glucose and lipid metabolism and immunity as valuable clues of intestinal maturity

**DOI:** 10.1186/s12864-017-4001-2

**Published:** 2017-08-22

**Authors:** Ying Yao, Valentin Voillet, Maeva Jegou, Magali SanCristobal, Samir Dou, Véronique Romé, Yannick Lippi, Yvon Billon, Marie-Christine Père, Gaëlle Boudry, Laure Gress, Nathalie Iannucelli, Pierre Mormède, Hélène Quesnel, Laurianne Canario, Laurence Liaubet, Isabelle Le Huërou-Luron

**Affiliations:** 1 Nutrition Metabolisms and Cancer (NuMeCan), INRA, INSERM, Université de Rennes 1, UBL, Rennes, Saint-Gilles France; 2 Animal Nutrition Institute, Sichuan Agricultural University, Chengdu, Sichuan China; 3 GenPhySE, Université de Toulouse, INRA, INPT, ENVT, Castanet Tolosan, France; 4 PEGASE, INRA, Agrocampus Ouest, Saint-Gilles, France; 5 Toxalim (Research Centre in Food Toxicology), Université de Toulouse, INRA, ENVT, INP-Purpan, UPS, Toulouse, France; 6 GenESI, UE1372, INRA, Surgères, France

**Keywords:** Maturity, Intestine, Lipid and glucose metabolism, Inflammation, Microarray, Pig

## Abstract

**Background:**

Maturity of intestinal functions is critical for neonatal health and survival, but comprehensive description of mechanisms underlying intestinal maturation that occur during late gestation still remain poorly characterized. The aim of this study was to investigate biological processes specifically involved in intestinal maturation by comparing fetal jejunal transcriptomes of two representative porcine breeds (Large White, LW; Meishan, MS) with contrasting neonatal vitality and maturity, at two key time points during late gestation (gestational days 90 and 110). MS and LW sows inseminated with mixed semen (from breed LW and MS) gave birth to both purebred and crossbred fetuses. We hypothesized that part of the differences in neonatal maturity between the two breeds results from distinct developmental profiles of the fetal intestine during late gestation. Reciprocal crossed fetuses were used to analyze the effect of parental genome. Transcriptomic data and 23 phenotypic variables known to be associated with maturity trait were integrated using multivariate analysis with expectation of identifying relevant genes-phenotypic variable relationships involved in intestinal maturation.

**Results:**

A moderate maternal genotype effect, but no paternal genotype effect, was observed on offspring intestinal maturation. Four hundred and four differentially expressed probes, corresponding to 274 differentially expressed genes (DEGs), more specifically involved in the maturation process were further studied. In day 110-MS fetuses, Ingenuity® functional enrichment analysis revealed that 46% of DEGs were involved in glucose and lipid metabolism, cell proliferation, vasculogenesis and hormone synthesis compared to day 90-MS fetuses. Expression of genes involved in immune pathways including phagocytosis, inflammation and defense processes was changed in day 110-LW compared to day 90-LW fetuses (corresponding to 13% of DEGs). The transcriptional regulator *PPARGC1A* was predicted to be an important regulator of differentially expressed genes in MS. Fetal blood fructose level, intestinal lactase activity and villous height were the best predicted phenotypic variables with probes mostly involved in lipid metabolism, carbohydrate metabolism and cellular movement biological pathways.

**Conclusions:**

Collectively, our findings indicate that the neonatal maturity of pig intestine may rely on functional development of glucose and lipid metabolisms, immune phagocyte differentiation and inflammatory pathways. This process may partially be governed by *PPARGC1A*.

**Electronic supplementary material:**

The online version of this article (doi:10.1186/s12864-017-4001-2) contains supplementary material, which is available to authorized users.

## Background

Mortality rate of young children is the best indicator of child health [1]. Despite a sharp reduction in the last 15 years, the total under-5 years mortality rate in the world was still as high as 5.9% of all viable fetuses in 2015 [1]. A recent analysis pointed out that late gestation to postnatal day 27 is the most at risk period compared to other age ranges considering its greatest relative probability of infant dying and its slowest decline from 2000 to 2015 [1]. Studying prematurity is a way to understand the important factors involved in survival. Preterm birth complications are the main causes for stillbirth and neonatal infant death, followed by inflammation, diarrhea, severe infections, and asphyxia [2]. Altered carbohydrate and lipid metabolisms were observed in preterm neonates [3, 4]. Moreover, increasing evidences suggest that impaired infection control could be attributed to underdeveloped immune system in preterm newborns [5–7]. Therefore delayed physiological maturity at birth compromises adaptation to extrauterine life, and accordingly impairs perinatal survival and child health.

The intestine, as the major organ for nutrient absorption and the largest immune organ, fulfills its maturation until 28–112 days of age in neonates [8]. The frequency and severity of necrotizing enterocolitis (NEC), the most common and deadly intestinal disease in neonatal intensive care units, increase with the degree of prematurity [9]. Though still poorly understood, existing data suggest that immaturity of the intestinal innate immune system associated with improper microbiota colonization is predisposing risk factor for NEC [10, 11]. Moreover, the intrauterine growth restriction (IUGR) that modifies the developmental pattern of the intestine is associated with increased neonatal morbidity and mortality, and worsens the risk of enteral feeding-induced NEC in preterm babies [12–14].

Fetal development of the intestine can be divided into three successful phases: cell proliferation and morphogenesis, cell differentiation and functional development [15]. The first two well-described phases that end at around 22 weeks of gestation in humans, result in a fetal intestine with a morphology close to that of the adult and with some functional capacities, related to the expression of digestive enzymes and nutrient transporters [16]. Functional acquisition of the mucosal immune system appears during mid-late gestation, mostly explaining the high infection risk in premature infants [17]. Insufficient vascularization and motility play some intermediary roles in NEC-associated pathologies in preterm infants, such as ischemia and pneumatosis [10, 18]. Moreover, no aboral transport of luminal content occurs in the intestinal tract of fetuses younger than 30 weeks of gestation while mature motor patterns are well established in term newborns [16]. A broad range of prenatal changes such as modification of endocrine secretion and intestinal functional capacities was reported to occur from day 90 of gestation up to term in pigs [19, 20]. Multiple time points during this late fetal stage were used to investigate the developmental pattern of intestinal functions in fetuses [14, 19], but these studies did not care about intestinal maturity. Thus, a more in-depth deciphering of the late developmental pattern of intestinal functions in individuals with different mortality rate at birth is a meaningful strategy to identify candidate biological pathways and regulators of intestinal maturity and may provide clues for perinatal survival and adequate adaptation to enteral feeding.

Because of the difficulty of getting human intestinal samples during late fetal development, an alternative to currently used intestinal cell lines and organ cultures is to take the opportunity offered by animal models such as the pig that share similar physiologic and clinical features with humans. The pig is increasingly used as a hypersensitive model for pediatric gastroenterology [8, 21, 22]. The Large White (LW) breed, genetically-selected for lean growth and prolificacy, displays a high rate of mortality at birth due to a lower physiological maturity of newborn piglets, whereas the Chinese Meishan (MS) breed produces piglets with extremely low mortality rate although lighter birth weight [23, 24]. We hypothesized that distinct developmental profiles of the fetal intestine during late gestation could contribute to the differences in neonatal maturity between the two breeds. Through transcriptomic analysis, our aim was to comprehensively clarify intestinal maturation with the expectation of finding relevant biological processes and regulators by using these two porcine breeds, divergent in neonatal morbidity and mortality, at two ages during the late fetal developmental stage. MS and LW sows were inseminated with mixed semen (from breed LW and MS). Thereby, each litter was composed of both purebred (LW or MS) and crossbred fetuses (LWMS from MS sows and MSLW from LW sows). This reciprocal design was used to investigate the possible impact of parental genomes on intestinal maturation. Finally transcriptomic data and 23 phenotypic variables (blood parameters, indicators of intestinal morphometry and digestive enzyme activities) already known to be associated with maturity trait were integrated using multivariate analysis in order to identify relevant sets of genes that correlate with changes in phenotypic variables.

## Methods

### Experiment design and animal sampling

The experimental design was previously described [25]. Briefly, nine MS and nine LW sows were inseminated with mixed semen (LW and MS) to get litters composed of both purebred (LW or MS) and crossbred fetuses (LWMS from MS sows and MSLW from LW sows). MS and LW breeds were chosen as two extreme breeds for piglet mortality at birth, a better survival rate being observed in MS piglets although they are lighter at birth. In addition, tissue developmental process was taken into account by sampling fetuses in two late fetal periods, at 90 and 110 days of gestation (duration of gestation is 114 days in swine). Therefore to determine changes in intestinal morphometry, enzyme activity and gene expression and in blood parameters during maturation, jejunum and umbilical cord blood were sampled from 63 porcine fetuses in eight different conditions: two gestational ages (day 90 and day 110) associated with four genotypes. All fetuses used in this study are from the experiment previously described by Voillet et al. [25] and were obtained by caesarean delivery. After laparotomy of the sow, piglets were individually exposed and blood was sampled separately from the umbilical artery and vein and plasma stored at −20 °C. After section of the umbilical cord, the fetus was euthanized with an intracardiac injection of 3 M potassium chloride and a middle segment of jejunum was sampled and placed into RNAlater (Applied Biosystems) at 4 °C for 24 h and then maintained at −20 °C until RNA extraction. For morphometry, a 1-cm adjacent jejunum sample was collected, rinsed with cold phosphate buffered saline and fixed in 4% paraformaldehyde for 24 h until serial dehydration in ethanol and embedding in paraffin. Finally, mucosa was scraped from an adjacent 50-cm segment, frozen in liquid nitrogen and stored at −80 °C until enzyme activity analysis. Three to five fetuses per litter were collected and weighted and their gender recorded. They were selected taking into account their body weight that was the closest to the mean body weight of each litter. These fetuses were genotyped with eight microsatellite markers, to discriminate the crossed breeds from the pure breeds. Thereby 80 male fetuses (10 fetuses per group) were selected. Finally 63 fetuses were analyzed for microarray experiment according to RNA quality. Blood parameters, intestinal morphometry and enzymes were also analyzed on these 63 fetuses.

### Analysis of blood parameters

Blood parameters were analyzed using standard methods listed in Additional file 1: Table S1. Glucose, fructose, lactate, albumin, cortisol, noradrenaline, adrenaline, dopamine, free and total thyroxine (T4), free and total triiodothyronine (T3), insulin like growth factor 1 (IGF1) were chosen as indicators of carbohydrate and protein metabolisms and hormonal regulation that are reported to be modified during the late gestational period and to differ according to genotypes [26, 27].

### Intestinal morphometry and enzyme activity analysis

Villous and crypt sizes were determined on haematoxylin-eosin-stained jejunum sections. A minimum of 15 well-oriented villus and 10 well-oriented crypts were measured under a light microscope (Nikon Eclipse E40, Nikon Instruments, France) using an image analysis software (NIS-Elements AR 3.0, Nikon Instruments). The specific activities of lactase and aminopeptidase N were determined in jejunum mucosa as previously described [28]. The protein concentration was measured according to the method of Lowry [29].

### Intestinal RNA extraction and microarray description

Total RNA was extracted and quantified from individual jejunum samples as previously described [30]. The porcine microarray (8 × 60 K, GPL16524, Agilent Technology) used consisted in 43,603 probes derived from the 44 K Agilent-026440 porcine specific microarray (V2, for 71% of total probes), 3773 probes from immune system, 9532 probes from adipose tissue and 3768 probes from muscle tissue, as already reported [25]. After quality control and quantity normalization steps, fluorescence signal data from 63 microarrays containing 39,446 spots were available for further statistical analysis and log2- transformed. Annotation was based on Voillet et al. (2014) data [25]. Nevertheless, all differentially expressed probes (DEPs) were double-checked and some probes were manually annotated by sequence blasting against NCBI or Ensemble (release 84) databases. Annotation was firstly blasted in NCBI and Ensemble against the *Sus scrofa* genome, and when necessary, successively against human, *Mus musculus* and *Rattus norvegicus* genomes when available. 90.2% of DEPs were annotated, raw data and information are available in NCBI (GEO accession number GSE90980).

### Quantitative Real-time PCR analysis of differentially expressed genes

The mRNA levels of DEGs between groups on microarrays were validated by relative quantitative Real-time PCR (qRT-PCR). Two micrograms of total RNA isolated from jejunum (*n* = 6, isolated for microarray analysis) was reverse transcribed using High capacity cDNA reverse transcription kit (Applied Biosystems). qRT-PCR analysis was performed using Fast SYBR Green technology on StepOnePlus system (Applied Biosystems). The primers were designed using Primer3 (NCBI, Primer-BLAST). When possible, primer pairs are located on exon-exon junction (Additional file 2: Table S2). Two-fold dilution series of cDNA from pooled samples were used to measure the specific amplification efficiency of each primer pair (99–104%). PCR cycle of amplification consisted of denaturation at 95 °C for 3 s and hybridization and extension at 60 °C for 30 s. Quantification cycle values of genes were converted into raw data with the highest expressed sample as a calibrator. The relative expressions of target genes were acquired after normalizing to the highly stable reference genes identified using GeNorm [31]. In this experiment, *PPIA* (peptidylprolyl isomerase A) and *TBP* (TATA box binding protein) were identified as stable reference genes. Pearson’s correlations were calculated between microarray values and qRT-PCR relative expressions.

### Statistical analysis

Analyses were performed using the R software version 3.1.3 [32].

Blood parameters, jejunum morphometry and enzyme activities were analyzed by a two-way analysis of variance (ANOVA) with gestational age (d90 and d110), genotype (LW, MSLW, LWMS and MS) and the interaction between gestational age and genotype as main fixed effects. Normality of data and homogeneity of variance were confirmed by Shapiro and Bartlett tests, respectively; data were Box-Cox transformed when their distribution was not normal. Models and procedures used to analyze transcriptomic data have been described in Voillet et al. [25]. A mixed linear model was applied to microarray data using two fixed effects (gestational age and fetal genotype), their interaction, and the sow as random effect, then compared to a reduced model by F-type test to identify DEPs for gestational age and/or fetal genotype. After correction by False Discovery Rate (FDR), the list of DEPs with a FDR-adjusted *P* value <0.01 was distributed into four sub-models using the Bayesian Information Criterion (BIC). Sub-model 1 combined the two fixed effects and their interaction (complete model); sub-model 2 involved the two fixed effects in an additive manner (additive model); sub-model 3 concerned only the effect of gestational age (gestational age model); and sub-model 4 included only the fetal genotype effect (genotype model). Moreover, to analyze the parental impact, a mixed linear model involving the two parental genotypes (maternal genotype and paternal genotype), the gestational age, and their interactions was applied to microarray data [25].

### Functional enrichment analysis and pathway analysis

Functional enrichment analysis of DEPs in purebred LW and MS was supplied by GeneCoDis 3.0 software [33] focused on Gene ontology (GO) Biological Process (BP), using the *Homo sapiens* genome (Ensembl 65 release) as reference. Functional enrichment analysis was applied on DEPs of the complete model with absolute log2-fold change ≥0.5 (corresponding to an absolute fold change 1.4) between day 90 and day 110. To set the statistical enrichment of a particular biological function, a hypergeometric test was used. All *P-*values were adjusted using the FDR approach (FDR *<* 0.01).

DEPs of the complete model in purebred LW and MS that meet the conditions described above (adjusted *P* value ≤0.01 and absolute log2-fold change ≥0.5) were subjected to Ingenuity® Pathway Analysis [34] (IPA; Ingenuity Systems, Qiagen, USA) for literature-based biological analysis. Standard settings of the *network* toolbox were applied with absolute log2-fold change ≥0.5, adjusted *P*-value ≤0.01 and direct relationships. The *Upstream Regulator Analysis* was performed to identify potential upstream transcriptional regulators and ligand-dependent nuclear receptors of differentially expressed genes. The Fisher’s exact test *P*-value was used to calculate the probability that the observed and predicted regulated gene sets were unlikely to be identified by chance alone. To evaluate the matching between observed and predicted up/down regulation patterns, activation z-scores were also determined.

### Multivariate integration and regression analysis

Multivariate statistics were used to select highly relevant genes that may predict phenotypic variable variation. We assessed the ‘uni-directional’ relationships between microarray dataset and phenotypic variables. The dataset used here including all expressed probes. For this purpose, the probes were considered as predictors of phenotypic variables using the sparse partial least square regression model (sPLS) implemented in mixOmics R package [35]. sPLS regression is related to classical PLS regression but it proceeds with additional variable selection that reduces noisy data and increases the predictive performance of the model, especially when the number of variables is far greater than the number of samples [36]. The number of genes selected in the first four components was tuned by Q2 criterion (> 0.09) to obtain the best predictive performance [37–39]. Mean square error of prediction (MSEP) and R2 were computed to assess the quality of prediction. With this approach, we finally retained the first component (Q2 = 0.21) and all gene-phenotypic variable relationship with an absolute correlation score > 0.75 to build a regression network with sPLS gene-phenotypic variable relationship. In order to provide a more biological integrative view of this model, we proceeded with a functional annotation of sPLS selected-genes using IPA *network* toolbox. Cytoscape (version 3.4.0) was used for network illustration.

## Results

### Intestinal morphometry and enzyme activities

**Fig. 1:**
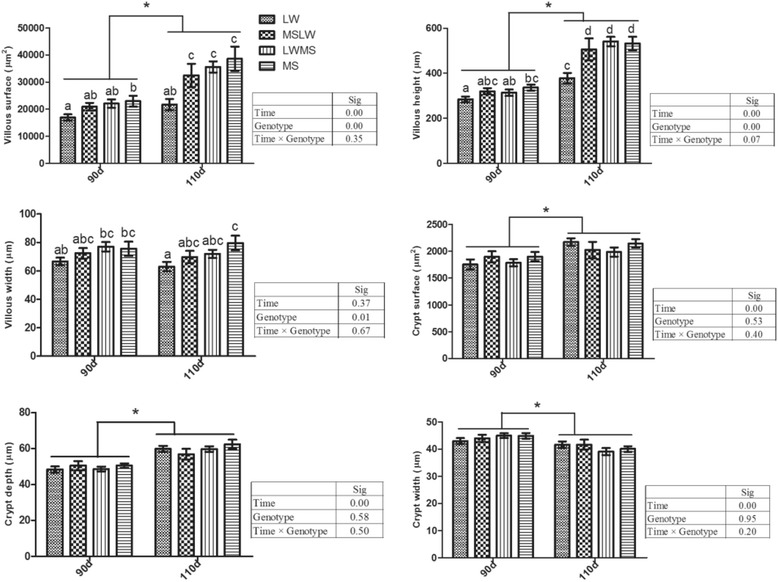
Jejunum morphometry in fetuses with different genotypes (Genotype) at 90 and 110 day of gestation (Time). Data are means ± SEM, *n* = 6–9/group. Means not sharing the same superscript letter differed significantly (*P* ≤ 0.05); * *P* ≤ 0.05 for the effect of gestational age (Time). LW, purebred fetuses from Large White sows; MSLW, crossbred fetuses from Large White sows; LWMS, crossbred fetuses from Meishan sows; MS, purebred fetuses from Meishan sows

**Fig. 2:**
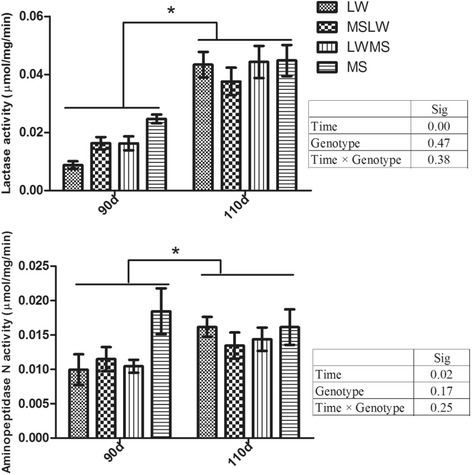
Jejunum lactase and aminopeptidase N activities in fetuses with different genotypes (Genotype) at 90 and 110 day of gestation (Time). Data are means ± SEM, *n* = 6–9/group. * *P* ≤ 0.05 for the effect of gestational age (Time). LW, purebred fetuses from Large White sows; MSLW, crossbred fetuses from Large White sows; LWMS, crossbred fetuses from Meishan sows; MS, purebred fetuses from Meishan sows

Villous surface and height increased with fetal age (*P* < 0.001) (Fig. 1). Purebred LW fetuses had lower villous surface and height compared to purebred MS at day 90 (*P* < 0.05) and compared to the other three genotypes at day 110 (*P* < 0.05). Irrespective of genotype, fetuses at day 110 had greater crypt surface and crypt depth and lower crypt width (*P* < 0.001), while the effects of genotype and the genotype × gestational age interaction were not significant (Fig. 1). Lactase and aminopeptidase N activity increased with age (*P* < 0.001), but were affected neither by genotype nor by the genotype × gestational age interaction (Fig. 2).

### Blood parameters

Fourteen blood metabolites and hormones were analyzed in both umbilical artery and vein. These parameters were chosen as indicators of metabolic changes that occur late in gestation. Similar trends in parameters between artery and vein were found (Additional file 1: Table S1). Briefly, most of the parameters differed with gestational age: albumin (12.4%; 12.8%), cortisol (208.6%; 152.1%), noradrenaline (76.0%; 135.7%), dopamine (89.7%; 98.3%), total T4 (26.9%) and free T4 (10.1%) increased between day 90 and day 110 of gestation while fructose (66.5%; 65.1%), lactate (42.2%; 39.8%) and adrenaline (82.4%; 62.2%) decreased during the same age period, in arterial and venous blood, respectively. Interestingly, blood fructose levels were 2.7–3.9 fold higher than glucose ones in fetuses at 90 days of gestation whereas differences were no more observed at 110 days of age, illustrating possibly crucial role of fructose to stimulate growth of porcine fetus [40, 41]. The effect of genotype was significant for fructose (crossbred fetuses from LW sows, MSLW, had the highest fructose in artery and vein while crossbred fetuses from MS sows, LWMS, had the lowest fructose), albumin (purebred MS fetuses had the highest albumin in artery and vein while purebred LW fetuses had the lowest albumin in artery and vein) and T4 (crossbred fetuses from LW sows, MSLW, had the highest total and free T4 while purebred LW fetuses had the lowest total and free T4). Finally the gestational age × genotype interaction was significant for lactate, albumin and T4.

### Influence of parental genotype on gene expression

**Fig. 3:**
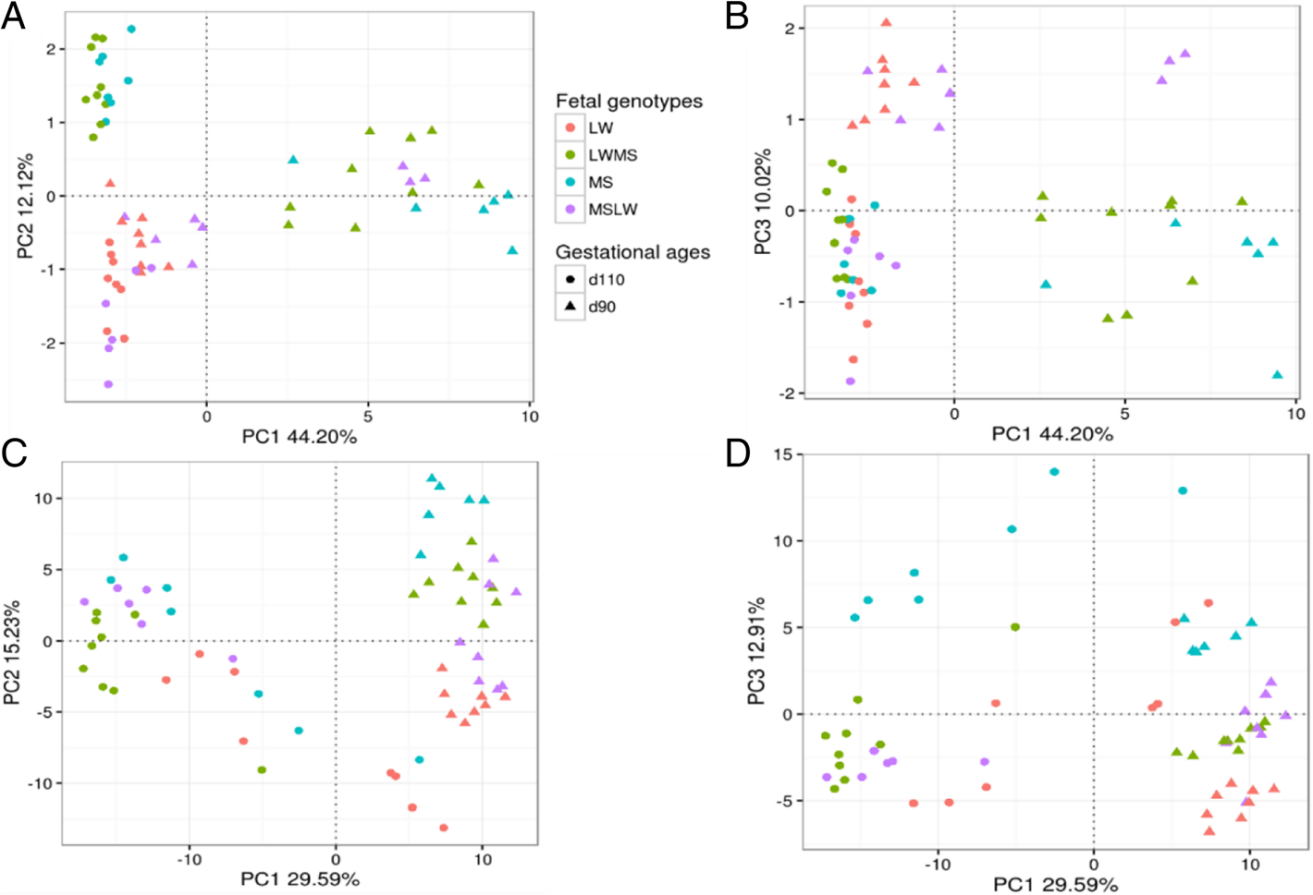
PCA of the maternal effect (**a**, **b**) and the complete model (**c**, **d**). LW, purebred fetuses from Large White sows; MSLW, crossbred fetuses from Large White sows; LWMS, crossbred fetuses from Meishan sows; MS, purebred fetuses from Meishan sows

Effects of paternal genotype or maternal genotype on intestinal maturation were detected by reciprocal crossing. Surprisingly, no probe was influenced by paternal genotype. Only 28 DEPs (corresponding to 18 unique differentially expressed genes, DEGs) were found as affected by maternal genotype in interaction with gestational age (FDR < 0.05, Additional file 3: Table S3). Principal component analysis (PCA) analysis of the 28 DEPs illustrated the moderate effects of the maternal genotype (PC1 = 44.2%, PC2 = 12.1% and PC3 = 10.0% of the total variance; Fig. 3a and b). However, PC1 mainly separated data from purebred MS fetuses at day 90 from those of purebred LW fetuses, PC2 separated data from fetuses from MS sows at day 110 from those of fetuses from LW sows, and PC3 separated data from fetuses from LW sows at day 90 from those of fetuses from MS sows at day 90.

**Fig. 4:**
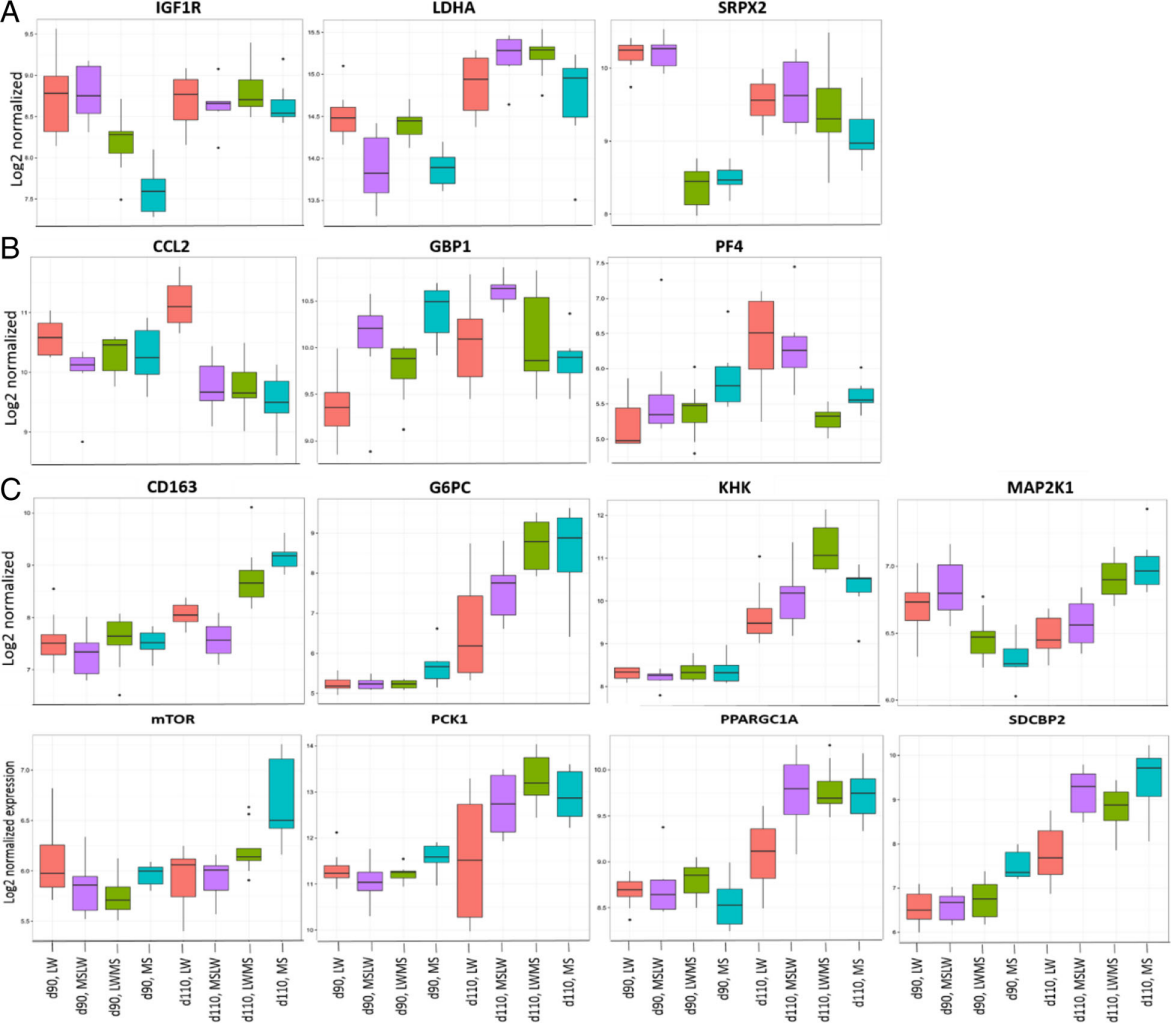
*Box-plot* representation of genes differentially expressed in fetuses with different genotypes at 90 (d90) and 110 day of gestation (d110). **a** Genes up-regulated in day 110-MS compared to day 90-MS. **b** Genes up-regulated in LW compared to MS at day 110. **c** Genes up-regulated in MS compared to LW at day 110. The gene expression was log2 transformed. LW, purebred fetuses from Large White sows; MS, purebred fetuses from Meishan sows

Eighty-two percent of the 28 DEPs were identified in the complete model (Additional file 3: Table S3), among which 11 unique DEGs were only differentially expressed in MS, with absolute log2-fold change ≥0.5, between day 110 and day 90. Spermine synthase (*SMS*) and *SRPX2* genes are specifically located on the X chromosome. As only male fetuses were chosen in this study, these two genes were of maternal origin. The expression profile of *SRPX2* gene (Fig. 4a) illustrated how the maternal effect is observed at 90 days of gestation. Functional analysis of these genes indicated that they are involved in activation of cell generation and differentiation, metabolism and angiogenesis.

Taken together, a barely effect of parental genome was found on offspring fetal intestinal maturation, and most of genes affected by the maternal genotype were identified in the complete model. Considering our aim was to decipher candidate biological pathway and genes that shape intestinal maturity, the complete model which combined joint differences between gestational age and fetal genotypes was therefore worthy of further analysis.

### Identification of intestinal DEPs

A total of 9518 probes was identified as differentially expressed (with a significant threshold of 1% with FDR correction) and divided into four sub-models using the BIC criterion (Additional file 4: Table S4). The complete model involved 404 DEPs corresponding to 274 unique DEGs. This smallest subset of DEPs was the most meaningful for analyzing the indices of intestinal maturity. In other words, these 274 genes were involved in late intestinal developmental process with differences between genotypes.

The use of PCA for each model enabled to assess and visualize the experimental design power. Concerning the complete model, PC1, PC2 and PC3 explained 29.6, 15.2 and 12.9% of the total variance and segregated fetuses according to their gestational ages and purebred genotypes (Fig. 3c and d showed a cluster with the data from fetuses at day 90 while a high dispersion of data from fetuses at day 110), illustrating the interaction between gestational age and genotype effects. The PCA results of other three sub-models were shown in Additional file 5: Figure S1.

### Ontological analysis of DEGs between both gestational ages and purebred genotypes

A functional enrichment analysis was performed on DEPs from the complete model to identify enriched biological processes (BP) during late fetal intestinal maturation using GeneCodis. Using the threshold of absolute log2-fold change ≥0.5, 80 DEPs (corresponding to 49 unique DEGs) were overexpressed at day 90 compared to day 110 and 161 DEPs (corresponding to 95 unique DEGs) were overexpressed at day 110 compared to day 90 (Additional file 4: Table S4). Overall the functional enrichment analysis revealed that dramatic changes occurred between gestational age day 90 and 110 (Additional file 6: Table S5). At 110 days of gestation, most of the induced genes were involved in hormone regulation and nutrient metabolism (e.g. lipid, nitrogen compound and nutrient metabolism, glucocorticoid and insulin signaling, gluconeogenesis and oxidation-reduction processes). In addition, axon guidance and immunity signaling (e.g. complement activation, chemotaxis, response to stress, blood coagulation and immune response) were also modulated during the late period of gestation. Conversely, genes whose expression was reduced at day 110 were included in the following biological pathways: SMAD protein signal transduction, xenobiotic metabolism, transmembrane transport, blood coagulation, regulation of cell proliferation and transcription, and proteolysis.

Genes involved in these enriched BPs were further analyzed separately according to each purebred genotype (Table 1). Interestingly, the number of up-regulated genes involved in glucose and lipid metabolic processes at day 110 was higher in MS than in LW. In contrast the number of up-regulated genes at day 90 was close between LW and MS for most of BPs. It is noteworthy that four genes, whose expressions were different according to gestational age, were oppositely expressed in LW and MS (Table 2). Sushi-repeat containing protein x-linked 2 (*SRPX2*), up-regulated in MS while down-regulated in LW at day 110 compared with day 90, was found as a promoter of angiogenesis and neuronal development. Conversely, the probes coding chemokine ligand 2 c-c motif (*CCL2*) and guanylate binding protein 1 (*GBP1*) genes involved in inflammation and defense process, were over-expressed in LW and under-expressed in MS at day 110 compared with day 90.

**Table 1:** Enriched GOBPs at day 90 or 110 in the two purebred genotypes

Day	Items	GOBP Terms	Genes
110	GO:0006957	complement activation	**C9** *VSIG4*
	GO:0034641	cellular nitrogen compound, metabolic process	*SMS BCKDHB*
	GO:0006094	gluconeogenesis	*PPARGC1A PCK1*
	GO:0008286	insulin receptor signaling pathway	*MTOR MAP2K1 IGF1R*
	GO:0032868	response to insulin stimulus	*MTOR PCK1*
	GO:0051384	response to glucocorticoid stimulus	*MAP2K1 BCKDHB*
	GO:0007584	response to nutrient	*MTOR BCKDHB*
	GO:0055114	oxidation-reduction process	*CYP3A4 ACOX2*
	GO:0006629	lipid metabolic process	**BTNL3** *APOD PCK1*
	GO:0006935	chemotaxis	**PF4** *MAP2K1*
	GO:0008380	RNA splicing	*CCAR1 RNPC3 PPARGC1A* **JMJD6**
	GO:0006805	xenobiotic metabolic process	*CYP3A4 CYP17A1*
	GO:0006950	response to stress	**HSPB8 HSPA8**
	GO:0007411	axon guidance	*MAP2K1 LGI1*
	GO:0007165	signal transduction	*PRKG2 MTOR MAP2K1* **FGL2** *IGF1R* **PDE11A** *RASAL2*
	GO:0007596, GO:0030168	blood coagulation, platelet activation	*PRKG2* **PF4 PDE11A**
	GO:0006955	immune response	*TCF12* **PF4** *IGHA1 IGF1R*
90	GO:0060395	SMAD protein signal transduction	**BMP2** *HNF4A*
	GO:0006805	xenobiotic metabolic process	**GSTA1** *CYP11A1 HNF4A ALDH2*
	GO:0055085, GO:0006811	transmembrane transport, ion transport	*KCNS3 AQP10* **SLC38A3**
	GO:0007596	blood coagulation	**ANXA8** *HNF4A*
	GO:0008285	negative regulation of cell proliferation	**LEPREL1 BMP2** *HNF4A*
	GO:0045893	positive regulation of transcription	**BMP2** *HNF4A*
	GO:0006508	proteolysis	**TPSD1 CPE**

In italic, genes are up-regulated in MS only, and in bold, genes are up-regulated in LW only. Genes up-regulated in both MS and LW are not represented (see details in Additional file 6: Table S5)

**Table 2:** Gestational age-affected probes with opposite expression between LW and MS

Gene	Probe	BH	Log2-Fold Change between day 110 and 90 in LW	Log2-Fold Change between day 110 and 90 in MS	Main GOBPs
CCL2	A_72_P412643	6.27E-07	0.562774745	−0.776799778	GO:0006954 inflammatory response
CCL2	A_72_P675364	3.57E-07	0.598172442	−0.755798549	GO:0006954 inflammatory response
GBP7	O10866	3.90E-06	0.713558263	−0.653564571	GO:0003924 GTPase activity
GBP1	gi|115,551,583|dbj|AK239047.1|	4.51E-05	0.697887594	−0.51343506	GO:0060333 interferon-gamma-mediated signaling pathway
SRPX2	A_72_P210412	1.00E-05	−0.635376842	0.6315505	GO:0001525 angiogenesis, GO:0051965 positive regulation of synapse assembly

**Fig. 5:**
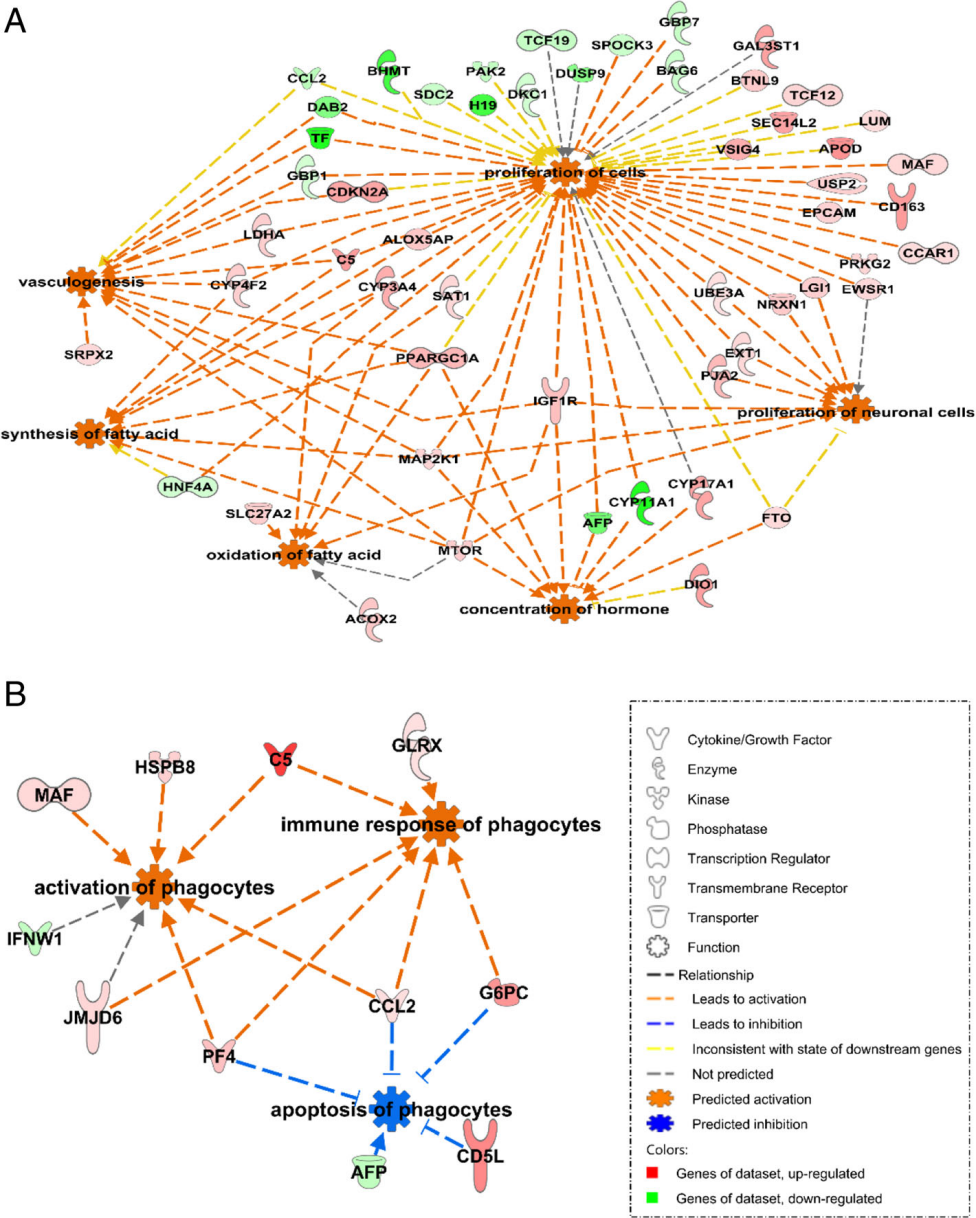
IPA network approach using Disease and Function analysis applied to DEGs in MS (**a**) and LW (**b**) at day 110 compared to day 90. Genes in *red* and *green* were up-regulated or down-regulated in the study dataset, respectively. A higher color intensity means the higher degree of up or down-regulation, respectively. LW, purebred fetuses from Large White sows; MS, purebred fetuses from Meishan sows

GOBP functional enrichment was further confirmed using disease and function analysis of IPA (Fig. 5; Additional file 7: Table S6). In day 110-MS compared to day 90-MS fetuses, pathways of fatty acid metabolism, hormone synthesis, cell proliferation, vasculogenesis and neurodevelopment were predicted to be activated. In LW, activation of phagocytes and their immune response and inhibition of their apoptosis were found at day 110 compared to day 90. Among these enriched genes, glucose 6 phosphatase catalytic subunit (*G6PC*), phosphoenolpyruvate carboxykinase 1 (*PCK1*), lactate dehydrogenase A (*LDHA*), peroxisome proliferator-activated receptor gamma coactivator 1 alpha (*PPARGC1A*), insulin like growth factor 1 (*IGF1*) receptor (*IGF1R*), mitogen activated protein kinase kinase 1 (*MAP2K1*), mammalian target of rapamycin (*mTOR*), *SRPX2* and *CD163* were up-regulated in MS compared to LW at day 110, while platelet factor 4 (*PF4*), *CCL2* and *GBP1* were up-regulated in LW compared to MS at day 110 (Fig. 4). Taken together, the distinct patterns of gene expression profiles and involved BPs in MS and LW genotypes at two key time points of gestational development illustrate major differences in intestinal maturation between purebred MS and LW fetuses.

### Identification of upstream regulators

**Fig. 6:**
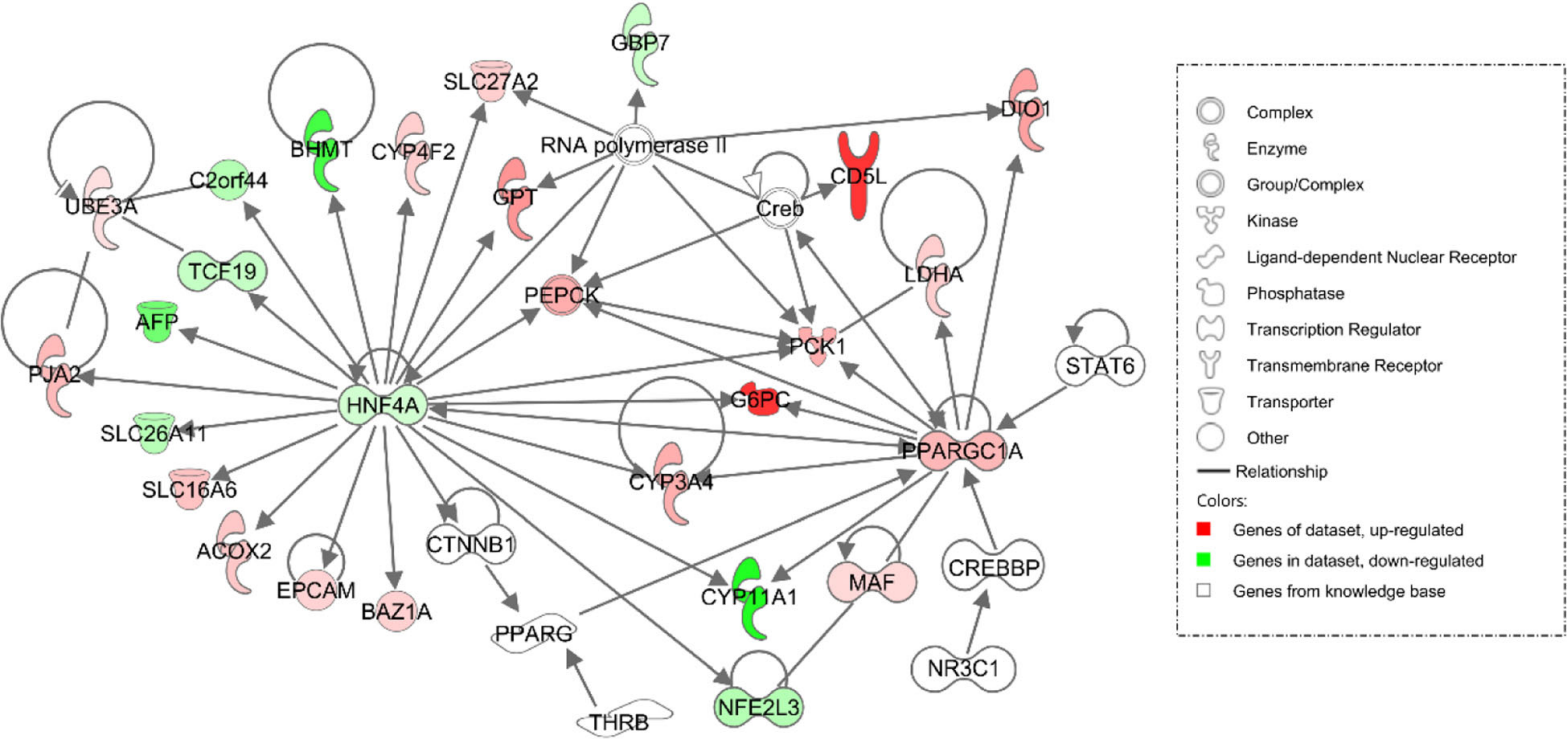
Network 1 (carbohydrate and lipid metabolism, energy production) in MS at day 110 compared to day 90 using IPA. Genes in *red* and *green* were up-regulated or down-regulated in the study dataset, respectively. A higher color intensity means the higher degree of up or down-regulation, respectively. Genes in white were from the knowledge base

We next thought to find upstream regulators that could explain the observed shifts in gene expression profiles and to identify candidate genes for prenatal intestinal maturation. Two ligand-dependent nuclear receptors and three transcriptional regulators were selected with an absolute activation z-score ≥ 2 in at least one genotype (Table 3). Nuclear receptor subfamily three group c member1 (*NR3C1,* also known as glucocorticoid hormone receptor, *GR*) and thyroid hormone receptor beta (*THRB*) were predicted to be active in both breeds though on a higher magnitude in MS, somehow illustrating similarity in upstream regulation between MS and LW genotypes. *NR3C1* was found in the fetal genotype model with a higher expression in LW compared to MS (Additional file 8: Figure S2) while *THRB* was found in the gestational age model with a decreased expression at day 110 compared to day 90. In addition to catenin beta 1 (*CTNNB1*) and signal transducer and activator of transcription 6 (*STAT6*), *PPARGC1A* was predicted to be active in day 110-MS fetuses. *PPARGC1A* (also known as *PGC1α*) the only upstream regulator from the complete model (Fig. 6) is known to be involved in regulation of fatty acid metabolism, vasculogenesis and hormone synthesis (Fig. 5a) as well as in gluconeogenesis. Taken together, *PPARGC1A* was identified as a potential important upstream regulator of several DEGs in day 110-MS fetuses.

**Table 3:** Upstream regulators of DEGs in purebred fetuses at day 110 compared to day 90

Upstream regulators	Activation z-score	
	LW	MS
NR3C1^a^	1.1	2.41
THRB^a^	1.2	2.18
*PPARGC1A* ^b^		2.23
CTNNB1^b^		2.18
STAT6^b^		2

^a^ligand-dependent nuclear receptors, ^b^Transcriptional regulators. In italic, the upstream regulator is expressed in the complete model. The upstream regulators with an absolute activation z-score ≥ 2 in MS or LW are only displayed

### Relationship between phenotypic variables and expression of genes

**Fig. 7:**
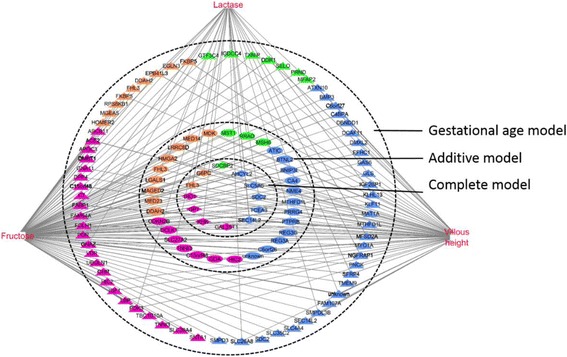
Network of relationships between phenotypic variables and microarray data selected within the first component of the sPLS analysis. Retained all gene-phenotypic variable pairs showing an absolute correlation score > 0.75. Related biological functions of selected genes was analyzed using IPA. *Red nodes*, phenotype items (blood fructose level, jejunum lactase activity and villous height); *pink symbols*, genes involved in lipid metabolism; *orange symbols*, genes involved in carbohydrate metabolism; *green symbols*, genes involved in cellular movement; *blue symbols*, other biological functions; *diamond shape symbol* indicates a differential expressed gene that was included in the complete model; *elipse symbol* indicates a differential expressed gene that was included in the additive model; *triangle symbol* indicates a differential expressed gene that was included in the gestational age model. sPLS, spare Partial Least Squares; IPA, Ingenuity Pathway Analysis

In the frame of complex systems biology, interactions between phenotypic and transcriptomic data can be explored using multivariate statistics. sPLS regression was used to assess the relationships between 23 phenotype items (seven indices of intestinal morphometry, two indices of intestinal digestive enzyme activity and 14 blood parameters) and gene expression. All expressed probes were used for analysis. Blood fructose level, intestinal lactase activity and villous height were the phenotypic variables best predicted by probes co-variation (mean squared error prediction = 0.28, 0.35 and 0.38, respectively). Functional analysis of these 450 probes (corresponding to 250 unique annotated genes) using IPA *network* identified lipid metabolism, carbohydrate metabolism and cellular movement as the top over-represented biological pathways (Fig. 7; Additional file 9: Table S7). Blood fructose level covaried with 15 genes involved in carbohydrate metabolism, 36 genes involved in lipid metabolism and 11 genes involved in cellular movement. Among them, seven genes identified in the complete model were positively (*FHL3*) or negatively (*G6PC*, *DIO1*, *KHK*, *GPT*, *GAL3ST1* and *SDCBP2*) correlated to blood fructose level. Similarly, lactase activity covaried with seven genes involved in carbohydrate metabolism, eight genes involved in lipid metabolism and two genes involved in cellular movement. Among them, four genes were identified in the complete model and were positively (*G6PC*, *DIO1* and *KHK*) or negatively (*FHL3*) correlated to lactase activity level. Finally villous height covaried with 11 genes involved in carbohydrate metabolism, 20 genes involved in lipid metabolism and four genes involved in cellular movement. Among them, seven genes that identified the complete model were positively (*G6PC*, *DIO1*, *KHK*, *GPT*, *GAL3ST1* and *SDCBP2*) or negatively (*FHL3*) correlated.

### Validation of differential gene expression by qRT-PCR analysis

To validate microarray results, seven differentially expressed genes of interest were selected to perform qRT-PCR. As shown in Table 4, the Pearson’s correlations between expression profiles of these genes measured by microarray and PCR was greater than 0.8 for all genes except *CIRBP.* The similarity of results between qRT-PCR and microarray confirmed the reliability of our microarray data (Additional file 10: Figure S3).

**Table 4:** Correlation between qRT-PCR expression and microarray data for selected genes

Genes	Pearson’s correlation	*P* value
CCL2	0.93	<0.001
CD163	0.83	<0.001
CIRBP	0.61	<0.001
FABP1	0.97	<0.001
GBP1	0.82	<0.001
G6PC	0.88	<0.001
PPARGC1A	0.82	<0.001

## Discussion

The use of a relevant experimental design (eight conditions in a two by four factorial design) leading to contrasting situations in terms of late fetal maturation associated with the selection of DEPs (FDR-adjusted *P* value <0.01) highlighted the impact of complex relationships between gestational age and fetal genotype on maturity of the intestine. No paternal genotype effect occurred while that of maternal genotype was moderate and mainly observed at 90 days of gestation. Overall 4794 unique DEGs (9518 DEPs, 24.1% of expressed probes of the microarray with FDR of 1%) were found valuable. The GO functional enrichment analysis revealed that the dramatic changes in intestinal gene expression that occurred between gestational age day 90 and day 110 concern genes involved in hormone regulation, nutrient metabolism, immune signaling and neuronal development. Furthermore, differences in the developmental pattern of the intestine were observed during late gestation between the two porcine breeds, divergent in neonatal morbidity and mortality and with higher survival rate in MS piglets. Lipid and glucose metabolisms, cell proliferation, vasculogenesis and neurodevelopment pathways were activated in the intestine of day 110-MS compared to day 90-MS fetuses whereas immune pathways including phagocytosis, inflammation and defense processes differed between day 110-LW and day 90-LW fetuses. *PPARGC1A* (also known as *PGC1α*) was identified as a potential upstream regulator of DEGs in MS. Finally the multivariate analysis indicated that blood fructose, and intestinal lactase activity and villous height were the three phenotypic parameters explained with the highest score by overlapping clusters of DEGs.

### Impact of parental genotype

Our experimental design included both pure breeds and the reciprocal crossed fetuses, allowing the exploration of the effect of paternal and maternal genotypes. No paternal genotype effect and only a moderate maternal genotype effect on intestinal gene expression (18 DEGs) were found in the current study. In contrast, a preferential paternal genotype effect was found on the gene expression in the piglet muscle from the same experiment [25], confirming that the parental genotype effect is a tissue-specific effect [42]. Thus, we focused on the complete model for identifying the main biological mechanisms of prenatal intestinal maturity.

### Biological implication of gene regulation in MS and LW during late gestation

Eighty percent of DEPs (7632 DEPs corresponding to 3866 DEGs) were influenced by the fetal gestational age (distributed among the gestational age model, the additive model and the complete model), demonstrating the great switch of gene expression that occurred between day 90 and day 110 of gestation. This was already observed in muscle development at the end of gestation in pig [25] and in sheep [43]. The wide changes in intestinal gene expression at the end of gestation confirm this gestational phase as a critical period for intestinal functional maturation. Accordingly 70% of DEGs that best explained the gestational age- and genotype-induced changes (first component of sPLS analysis) of the first three selected phenotype parameters (blood fructose, intestinal lactase activity and villous height) in the multivariate analysis were included in the gestational age model. Similarly, functional maturation of the fetal intestine occurs during mid-late gestation in humans [16]. Indeed most of the digestive enzymes, nutrient transporters and molecules involved in lipid and glucose metabolism measured in the human fetal intestine begin to be detectable shortly after the period of morphogenesis and increase with gestational age [16, 44].

The gestational age-induced developmental pattern of genes involved in glucose and lipid metabolism discriminated the two porcine genotypes, providing further evidences that these key genes and biological functions are involved in maturation of piglet intestine. Body energy reserves, i.e. glycogen and fat, are important predisposing factors involved in the maturation of liver and muscle [45, 46]. Muscle transcriptomic analysis has previously underlined the higher ability of MS fetuses to store and mobilize glycogen, known to be essential for early postnatal survival [25]. Consistent with these data, the expression of *G6PC*, *PCK1* and *LDHA* was increased in the intestine of day 110-MS fetuses (Fig. 4a and c). In preterm piglets the intestinal expression of *LDHA* was previously reported to be down-regulated [47]. The gene *PPARGC1A* (or *PGC1ɑ)* is a transcription coactivator that modulates metabolic pathways after binding to different transcription factors [48, 49]. The knockdown of *PGC1ɑ* in mice almost abolishes the expression of *PCK1* and *G6PC* [50]. Insulin and IGFs by binding to IGF1R exert multiple mitogenic effects through activation of numerous signaling pathways like phosphaitidylinositol 3 kinase (PI3K) and MAPK. mTOR belongs to the PI3K related kinase family, integrates signals from growth factors, nutrients, energy and stress (hypoxia, DNA damage) to induce metabolism response and regulate the cell cycle that is required for embryonic development [51]. Therefore the increased expression of both *PPARGC1A* and targeted genes such as *mTOR*, *IGF1R* and *MAP2K1*, exclusively in day 110-MS fetuses (Fig. 4a and c) could explain the activated glucose and lipid metabolic processes and cell proliferation in MS at day 110 of gestation and participate to the better survival rate of MS neonates. A greater energy reserve would better meet the high demand of the intestine for growth and functions at birth, and thereby potentially promote mucosal barrier development in agreement with the higher villous size measured in the intestine of day 110-MS fetuses. In addition the specific up-regulation of *SRPX2* and *PPARGC1A* expression in MS between day 90 and day 110 would promote angiogenesis thereby increasing nutrient supply to other organs, which is good for survival. Defaults in intestinal vasculature are also risk for postnatal enteral feeding in preterm neonates [18]. Overall one valuable key for maturity of the intestine could be a well-developed epithelial barrier and its vascularization associated with activated metabolic processes that provide energy reserves for functioning of other organs.

Another major difference between the two genotypes was the developmental pattern of the intestinal immune system. In contrast to MS, genes up-regulated at day 110 of gestation in LW are involved in phagocyte activation and inflammation (*PF4*, *CCL2* (also known as monocyte chemoattractant protein, *MCP1*) and *GBP1*) (Fig. 4b). PF4 is released from activated platelets, acts to facilitate blood clotting and plays a key pro-inflammatory role in inflammatory bowel disease and NEC [17, 52]. CCL2 is a chemokine secreted by monocytes, macrophages and endothelial cells; a higher CCL2 secretion was reported in immature human intestine compared to mature xenograft [9]. GBP1, induced by interferon, is involved in early activation of immune response to pathogen infection [53]. Moreover a lower expression of *CD163* (Fig. 4c) and transforming growth factor beta 2 (*TGFB2*) (Additional file 8: Figure S2) was observed in day 110-LW fetuses compared to day 110-MS. CD163 is a differentiation marker of the macrophage lineage with increased expression along the differentiation process [54]. Pro-inflammatory monocytes from blood differentiate into non-inflammatory intestinal resident macrophages when stimulated by TGFB2 [55, 56]. Therefore a lower *CD163* and *TGFB2* expression in day 110-LW may indicate less mature monocytes in LW intestine. TGFB2 also improves immune development of immature intestinal epithelial cells, thereby preventing their excessive inflammatory response [57–59]. Expression and activity of TGFB2 were decreased in intestinal tissues from NEC patients [55]. In our study *TGFB2* expression decreased between day 90 and day 110, but to a higher extent in LW. In contrast the expression and activity of TGFB2 in the intestine was reported to increase with advanced gestational age in humans and pigs, even though TGFB2 level is still low in human babies and pig neonates [17, 55, 60]. Further exploration is needed for understanding this discrepancy. The differentiation of maturated pro-inflammatory monocytes into intestinal resident macrophages and the progressive suppression of inflammation in intestinal epithelium cells are crucial for neonatal intestinal homeostasis and bacterial tolerance [17]. Overall the defective intestinal immune capacity associated with a higher pro-inflammatory status may indicate a lower immune defense ability and a higher risk of inflammatory response to environmental pathogens in LW neonates, that may participate to the higher morbidity and mortality scores reported in LW compared to MS neonates.

### Upstream regulators for prenatal intestinal maturity

IPA analysis revealed five upstream regulators with z-score ≥ 2. *NR3C1* (also known as glucocorticoid receptor, *GR*), with a higher z-score in day 110-MS than in day 110-LW fetuses, is involved in major physiological functions, including metabolism, cell growth and differentiation and immunity [61]. Glucocorticoids are well known to be involved in the late gestation to prepare organs to extra-uterine environment [45]. Serum cortisol level was reported to be higher and total *GR* expression in the liver was lower in newborn Erhualian piglet (a Chinese indigenous breed, close to MS) compared to LW piglet [62]. Consistently, similar breed differences in intestinal *NR3C1* expression were observed between MS and LW at day 110 (Additional file 8: Figure S2). *PPARGC1A* upstream regulator also displays a high activation z-score in MS and its expression is higher in day 110-MS compared to day 110-LW fetuses (Fig. 4c). In addition *PPARGC1A* is related to other upstream regulators and target genes of *PPARGC1A* were found to be consistently induced, making it the most interesting upstream regulator retrieved in our study. PPARGC1A has been reported to primarily regulate energy metabolism, especially fatty acid metabolism and gluconeogenesis, according to the nutrient/hormonal environment [48, 50]. In addition, activation of PPARGC1A is also of interest as it has being documented to coordinate the expression of anti-inflammatory properties and vascularization [49].

### Integration of blood and intestinal phenotypic variables and intestinal transcriptome

To get insight into the relationships between phenotype items and gene expression and to identify the key variables which can explain the biological outcomes of interest, sPLS was used to combine the phenotypic and microarray datasets without considering the fixed effects of fetal genotype and gestational age. Our correlation analysis revealed strong relationships between jejunum gene expression patterns and three phenotypic variables (blood fructose level, intestinal lactase activity and intestinal villous height). Piglet genotype did not modify the gestational age-induced increase in lactase activity, whereas it tended to modify the gestational age-induced changes in villous height (increase between day 90 and day 110) and blood fructose level (decrease between day 90 and day 110). Indeed, at day 110, higher values for villous height were observed in purebred MS and crossbred fetuses compared to purebred LW and the lowest values of fructose level were observed in crossbred MS and purebred MS. Blood fructose level was mostly correlated with genes involved in carbohydrate and lipid metabolisms, such as ketohexokinase (*KHK*), deiodinase 1 (*DIO1*) and *G6PC* (Fig. 7). Because of physiological differences in placentation between the two species, fructose is the primary hexose in the fetal pig blood but not in that of human [27]. Recently, fructose was demonstrated to stimulate trophectoderm cell proliferation and hyaluronic acid production, as did glucose, promoting the growth and development of the conceptus [63]. In addition, hyaluronic acid is an important promoter of embryonic vasculogenesis [64] and fructose has a better ability to stimulate anabolic metabolism than glucose in hepatocyte and cardiomyocytes through the action of KHK [65]. Therefore, the higher expression of genes involved in energy metabolism associated with the lower fructose level in day 110-MS fetuses may indicate advanced intestinal maturity in MS compared to LW piglets. Our data corroborates a recent metabolomics analysis in human neonates showing that insulin-dependent metabolism was inhibited in preterm compared to term babies [3]. Villous height was previously used as an intestinal maturity marker; the increasing villous height with fetal age means a greater absorptive area for enteral nutrition at birth [16]. Covarying with villous height, up-regulated expression of genes involved in carbohydrate and lipid metabolisms as well as in cellular movement such as *G6PC*, *KHK* and syndecan binding protein (or syntenin) 2 (*SDCBP2*), was observed in day 110-MS fetuses (Figs. 4c and 7). As discussed above, a higher metabolic activity could provide nutrient and energy for enterocyte proliferation and intestinal development. Moreover, *SDCBP2* was proved to affect cell survival and cell division through its high affinity to plasma membrane and nuclear phosphatidylinositol 4,5-bisphosphate [66]. Finally it is well known that increase in lactase expression is concurrent with the fetal increase in duodenal villi size and peaks at birth to handle milk lactose digestion [16]. Consistently, the gestational age-induced lactase activity was correlated with genes involved in energy metabolism. It is noticeable that the number of genes correlated with lactase activity changes and identified in the complete model was much lower than those correlated with blood fructose and villous height variables, suggesting that fetal intestinal lactase development is predominately an intrinsic age-related program [67]. Taken together, the three phenotypic variables highlighted in the current study were remarkably explained by clusters of overlapping DEGs. The additional analysis of phenotypic variables related to the mucosal immune system warrants further investigations.

## Conclusion

The results of this exploratory study provide a comprehensive overview of prenatal intestinal maturity. Considerable transcriptomic changes occurred during late gestation and were genotype-dependent. Improved epithelial structure and anabolic metabolism in day 110-MS fetuses highlighted the importance of mature intestinal metabolic capacities in neonates, corroborating the fact that metabolic dysfunction is associated with higher mortality risks in preterm infants. Blood fructose playing a great role in carbohydrate and lipid metabolism during late gestation was identified as a valuable clue of intestinal maturity and we propose that *PPARGC1A*, as an upstream gene regulator, may be a useful target to evaluate it. On the other hand, activated phagocytes and inflammatory response in day 110-LW fetuses may correlated to the delay in intestinal differentiation of macrophages and epithelial cells during late intestinal maturation. The expression profiles of genes related to immunity (*PF4, CCL2, GBP1, CD163 and TGFB2*) that could be valuable indicators of intestinal immune maturity in neonates, warrant further investigations with validation in the immature intestine of preterm newborns or neonates with intra-uterine growth retardation.

## Additional files

Additional file 1: Table S1. Concentration of blood parameters from umbilical cord of purebred fetuses (LW or MS) and crossbred fetuses (LWMS and MSLW) at 90 and 110 days of gestation [68–71]. (PDF 351 kb)

Additional file 2: Table S2. Primer sequences used for qRT-PCR. PPIA, peptidylprolyl isomerase A; TBP, TATA box binding protein; CCL2, C-C motif chemokine ligand 2; CIRBP, cold inducible RNA binding protein; FABP1, fatty acid binding protein 1; GBP1, guanylate binding protein 1; G6PC, Glucose 6 phosphatase; PPARGC1A, peroxisome proliferator-activated receptor gamma coactivator 1-alpha. (PDF 160 kb)

Additional file 3: Table S3. Complete list of DEPs depending on parental genotype effect. (XLSX 13 kb)

Additional file 4: Table S4. Complete list of DEPs in the four sub-models. The complete model involved 404 DEPs corresponding to 274 unique DEGs. The additive effect of gestational age and genotype was observed for 2871 DEPs corresponding to 1771 unique DEGs (30.2%). The gestational age effect that led to 4357 DEPs corresponding to 2462 unique annotated genes was the largest portion of DEPs (45.8%) while the effect of the fetal genotype included 1886 DEPs corresponding to 1251 unique DEGs (19.8%). (XLSX 898 kb)

Additional file 5: Figure S1. PCA analysis of the additive model, the gestational age model and the fetal genotype model. (a) For the gestational age model, the PC1 explained 30.0% of the total variance and segregated data from fetuses according to their age. (b) For the genotype model, the data from purebred LW and MS fetuses were clearly separated along the PC1 (24.3%) while the data from crossbred fetuses segregated on the PC3 (10.5%). (c) The gestational age segregation was also clear cut in PC1 (24.4%) of the additive model, while the purebred fetuses segregated on PC2 (16.2%). (PDF 153 kb)

Additional file 6: Table S5. Complete list of enriched GOBPs of four sub-models. The top 10 enriched biological pathways in the additive model were protein metabolism (cellular protein metabolism, protein folding and protein phosphorylation), cell cycle (mitotic cell cycle, cell division and apoptosis), blood coagulation, gene expression and cell adhesion. The top 10 enriched biological pathways of DEPs in the gestational age model were predominantly associated with protein metabolism, cell division (mRNA and RNA metabolism, apoptosis and anti-apoptosis, translation), blood coagulation, endocrine development and immunity. The top 10 enriched biological pathways in the fetal genotype model were mainly involved in cell cycle (cell cycle, cell division, negative regulation of apoptosis and chromatin modification), carbohydrate metabolism, immunity (cytokine signaling, interferon-gamma signaling), protein transport (protein transport and transmembrane transport) and blood coagulation. (XLSX 130 kb)

Additional file 7: Table S6. IPA Disease and function analysis of DEGs in MS and LW at day 110 compared to day 90. (XLS 37 kb)

Additional file 8: Figure S2. Box-plot representation of NR3C1 and TGFB2 expression in fetuses with different genotypes at 90 (d90) and 110 day (d110) of gestation. (PDF 120 kb)

Additional file 9: Table S7. Relationships between phenotypic variables and microarray data: list of genes selected within the first component of the sPLS analysis. Retained all gene-phenotypic variable pairs showed an absolute correlation score > 0.75. Related biological functions of selected genes was analyzed using IPA. (XLSX 36 kb)

Additional file 10: Figure S3. Box-plot representation of the seven tested genes in qPCR compared to their microarray expression. (PDF 359 kb)

## Abbreviations

CCL2: Chemokine ligand 2, c-c motif
CTNNB1: Catenin beta 1
DEGs: Differentially expressed genes
G6PC: Glucose 6 phosphatase catalytic subunit
GBP1: Guanylate binding protein 1
IGF1R: Insulin like growth factor 1 receptor
IUGR: Intrauterine growth restriction
KHK: Ketohexokinase
LDHA: Lactate dehydrogenase A
LW: Large White
MAP2K1: Mitogen activated protein kinase kinase 1
MS: Meishan
mTOR: mammalian target of rapamycin
NEC: Necrotizing enterocolitis
NR3C1: Nuclear receptor subfamily 3 group c member1
PCK1: Phosphoenolpyruvate carboxykinase 1
PF4: Platelet factor 4
PI3K: Phosphatidylinositol 3 kinase
PPARGC1A: Peroxisome proliferator-activated receptor gamma, coactivator 1 alpha
SDCBP2: Syndecan binding protein 2
SMS: Spermine synthase
sPLS: spare Partial Least Squares
STAT6: Signal transducer and activator of transcription 6
SRPX2: Sushi-repeat containing protein x-linked 2
TGFB2: Transforming growth factor beta 2
THRB: Thyroid hormone receptor beta
