# Supplementary material for: Comparing the intestinal transcriptome of Meishan and Large White piglets during late fetal development reveals genes involved in glucose and lipid metabolism and immunity as valuable clues of intestinal maturity
Source: BMC Genomics. 2017 Aug 22;18:647. doi: 10.1186/s12864-017-4001-2 (PMC5568345; doi:10.1186/s12864-017-4001-2)
Supplement: Supplementary file 1 — Concentration of blood parameters from umbilical cord of purebred fetuses (LW or MS) and crossbred fetuses (LWMS and MSLW) at 90 and 110 days of gestation [68–71]. (PDF 351 kb) [file 12864_2017_4001_MOESM1_ESM.pdf]

Additional file 1: Table S1. Blood parameter concentration from umbilical artery and vein of purebred fetuses (LW or MS) and crossbred fetuses (LWMS and MSLW) at 90 days and 110 days of gestation

Gestational age (days)	**90**				**110**				***P*-level**			**Method used for analysis**
	**LW**	**MSLW**	**LWMS**	**MS**	**LW**	**MSLW**	**LWMS**	**MS**	**T**	**G**	**T × G**	
Arterial glucose, mmol/L	1.87 ± 0.19	2.02 ± 0.19	2.12 ± 0.26	2.27 ± 0.17	2.51 ± 0.16	2.64 ± 0.33	2.00 ± 0.26	2.14 ± 0.22	0.14	0.78	0.16	Enzymatically (Glucose RTU kit: #61269, Biomérieux, Marcy l'étoile, France) [68]
Venous glucose, mmol/L	1.98 ± 0.22	2.01 ± 0.23	2.25 ± 0.22	2.58 ± 0.27	2.72 ± 0.22	2.94 ± 0.27	2.17 ± 0.22	2.36 ± 0.25	0.06	0.06	0.06	Enzymatically (Glucose RTU kit: #61269, Biomérieux, Marcy l'étoile, France) [68]
Arterial fructose, mmol/L	6.25 ± 0.57^{cd}	7.93 ± 0.51^{e}	5.77 ± 0.62^{c}	7.24 ± 0.48^{de}	2.77 ± 0.48^{ab}	3.13 ± 0.58^{b}	1.39 ± 0.18^{a}	1.83 ± 0.13^{ab}	<0.01	<0.01	0.07	Enzymatically (D-Fructose kit: #984302, Thermo Fisher Scientific, Vantaa, Finland) [68]
Venous fructose, mmol/L	5.96 ± 0.44^{bc}	7.58 ± 0.47^{c}	5.79 ± 0.44^{bc}	7.25 ± 0.54^{c}	2.81 ± 0.44^{a}	3.22 ± 0.54^{ab}	1.39 ± 0.44^{a}	1.85 ± 0.50^{a}	<0.01	<0.01	0.10	Enzymatically (D-Fructose kit: #984302, Thermo Fisher Scientific, Vantaa, Finland) [68]
Arterial lactate, mmol/L	2.31 ± 0.11^{ab}	2.72 ± 0.18^{bc}	4.08 ± 0.76^{cd}	4.96 ± 1.12^{d}	2.96 ± 0.41^{bc}	2.51 ± 0.38^{ab}	1.57 ± 0.12^{ab}	1.07 ± 0.11^{a}	<0.01	0.57	<0.01	Enzymatically (Lactate PAP kit: #61192, Biomérieux, Marcy l'étoile, France) [68]
Venous lactate, mmol/L	2.50 ± 0.41^{bc}	2.78 ± 0.44^{bc}	4.10 ± 0.41^{c}	4.92 ± 0.50^{c}	2.93 ± 0.41^{bc}	2.71 ± 0.50^{bc}	1.73 ± 0.41^{ab}	1.25 ± 0.47^{a}	<0.01	0.46	<0.01	Enzymatically (Lactate PAP kit: #61192, Biomérieux, Marcy l'étoile, France) [68]
Arterial albumin, g/L	5.13 ± 0.24^{a}	6.93 ± 0.25^{bc}	6.26 ± 0.42^{bc}	6.46 ± 0.52^{bc}	6.42 ± 0.34^{bc}	5.95 ± 0.30^{ab}	7.19 ± 0.24^{c}	8.41 ± 0.42^{d}	<0.01	<0.01	<0.01	Colorimetric (Albumin Kit: #6105, Biomérieux, Marcy l'étoile, France) [68]
Venous albumin, g/L	4.73 ± 0.25^{a}	6.29 ± 0.42^{b}	6.04 ± 0.37^{b}	7.47 ± 0.24^{c}	5.92 ± 0.34^{b}	5.82 ± 0.28^{b}	7.31 ± 0.29^{c}	8.51 ± 0.30^{d}	<0.01	0.04	0.04	Colorimetric (Albumin Kit: #6105, Biomérieux, Marcy l'étoile, France) [68]
Arterial cortisol, ng/mL	21.04 ± 2.66	15.27 ± 3.16	17.56 ± 1.76	17.01 ± 2.85	44.01 ± 6.10	39.92 ± 4.17	61.35 ± 14.63	73.44 ± 13.52	<0.01	0.38	0.49	Radioimmunoassay [69]
Venous cortisol, ng/mL	21.26 ± 5.82	14.78 ± 6.18	20.06 ± 6.18	17.59 ± 7.14	41.08v 5.82	35.51 ± 7.14	46.16 ± 5.82	63.05 ± 6.60	<0.01	0.10	0.34	Radioimmunoassay [69]
Arterial noradrenaline, ng/mL	2.51 ± 0.64	1.06 ± 0.33	2.37 ± 0.60	0.63 ± 0.17	3.83 ± 0.74	1.95 ± 0.49	2.34 ± 0.58	3.45 ± 0.85	<0.01	0.06	0.12	HPLC assay with electrochemical detection [70] after solvent extraction [71]
Venous noradrenaline, ng/mL	1.12 ± 0.32	0.71 ± 0.36	0.96 ± 0.34	0.68 ± 0.48	2.93 ± 0.34	1.61 ± 0.43	1.48 ± 0.36	2.14 ± 0.36	<0.01	0.09	0.67	HPLC assay with electrochemical detection [70] after solvent extraction [71]
Arterial adrenaline, ng/mL	1.05 ± 0.56	1.08 ± 0.49	0.91 ± 0.13	2.33 ± 1.84	0.61 ± 0.44	0.10 ± 0.02	0.10 ± 0.03	0.13 ± 0.02	<0.01	0.86	0.37	HPLC assay with electrochemical detection [70] after solvent extraction

												[71]
Venous adrenaline, ng/mL	0.15 ± 0.09	0.38 ± 0.11	0.27 ± 0.12	0.24 ± 0.19	0.14 ± 0.10	0.11 ± 0.13	0.08 ± 0.15	0.08 ± 0.12	0.04	0.76	0.54	HPLC assay with electrochemical detection [70] after solvent extraction [71]
Arterial dopamine, ng/mL	0.21 ± 0.03	0.18 ± 0.03	0.18 ± 0.05	0.62 ± 0.35	0.49 ± 0.16	0.29 ± 0.03	0.61 ± 0.20	0.89 ± 0.35	<0.01	0.07	0.40	HPLC assay with electrochemical detection [70] after solvent extraction [71]
Venous dopamine, ng/mL	0.19 ± 0.11	0.18 ± 0.12	0.16 ± 0.14	0.40 ± 0.19	0.33 ± 0.12	0.27 ± 0.15	0.47 ± 0.12	0.76 ± 0.14	0.01	0.03	0.44	HPLC assay with electrochemical detection [70] after solvent extraction [71]
Arterial total T4, µg/dL	3.70 ± 0.15^{a}	5.10 ± 0.16^{bc}	4.68 ± 0.14^{ab}	5.10 ± 0.29^{bc}	5.92 ± 0.50^{c}	5.87 ± 0.50^{c}	5.64 ± 0.37^{bc}	6.14 ± 0.43^{c}	<0.01	0.02	0.05	Automated immunoassay (AIA-360, Tosoh Bioscience, San Francisco, CA)
Arterial free T4, µg/dL	0.98 ± 0.05^{a}	1.40 ± 0.04^{b}	1.35 ± 0.03^{b}	1.38 ± 0.04^{b}	1.48 ± 0.10^{b}	1.44 ± 0.15^{b}	1.27 ± 0.06^{b}	1.44 ± 0.06^{b}	0.01	0.01	<0.01	Automated immunoassay (AIA-360, Tosoh Bioscience, San Francisco, CA)
Arterial total T3, ng/dL	22.73 ± 0.99^{a}	26.87 ± 2.12^{ab}	38.56 ± 8.87^{abc}	53.38 ± 11.16^{c}	38.74 ± 3.08^{abc}	35.17 ± 3.54^{abc}	44.98 ± 4.90^{bc}	39.61 ± 5.16^{abc}	0.08	0.07	0.07	Automated immunoassay (AIA-360, Tosoh Bioscience, San Francisco, CA)
Arterial free T3, ng/dL	0.58 ± 0.02	0.73 ± 0.08	1.16 ± 0.31	1.41 ± 0.40	0.96 ± 0.09	0.85 ± 0.10	0.97 ± 0.12	0.93 ± 0.12	0.90	0.26	0.21	Automated immunoassay (AIA-360, Tosoh Bioscience, San Francisco, CA)
Arterial IGF1, ng/mL	31.68 ± 3.31	24.31 ± 3.19	25.40 ± 1.95	21.56 ± 1.81	29.36 ± 4.06	26.08 ± 2.95	26.71 ± 1.89	33.17 ± 6.20	0.25	0.37	0.23	IRMA IGF-1 kit [68]

Within a same row, means (SEM) (n=6-9/group) not sharing the same superscript letter differ significantly ($P \leq 0.05$) for the effects of gestational age (Time, T) and of genotype (G)

LW, purebred fetuses from Large White sows; MSLW, crossbred fetuses from Large White sows; LWMS, crossbred fetuses from Meishan sows; MS, purebred fetuses from Meishan sows
