# Supplementary material for: Comparing the intestinal transcriptome of Meishan and Large White piglets during late fetal development reveals genes involved in glucose and lipid metabolism and immunity as valuable clues of intestinal maturity
Source: BMC Genomics. 2017 Aug 22;18:647. doi: 10.1186/s12864-017-4001-2 (PMC5568345; doi:10.1186/s12864-017-4001-2)
Supplement: Supplementary file 2 — Primer sequences used for qRT-PCR. PPIA, peptidylprolyl isomerase A; TBP, TATA box binding protein; CCL2, C-C motif chemokine ligand 2; CIRBP, cold inducible RNA binding protein; FABP1, fatty acid binding protein 1; GBP1, guanylate binding protein 1; G6PC, Glucose 6 phosphatase; PPARGC1A, peroxisome proliferator-activated receptor gamma coactivator 1-alpha. (PDF 160 kb) [file 12864_2017_4001_MOESM2_ESM.pdf]

Additional file 2: Table S2. Primer sequences used for qRT-PCR.

PPIA, peptidylprolyl isomerase A; TBP, TATA box binding protein; CCL2, C-C motif chemokine ligand 2; CIRBP, cold inducible RNA binding protein; FABP1, fatty acid binding protein 1; GBP1, guanylate binding protein 1; G6PC, Glucose 6 phosphatase; PPARGC1A, peroxisome proliferator-activated receptor gamma coactivator 1-alpha.

Gene names	Accession number	Sequence of primers, 5' -3'	
		Forward	Reverse
PPIA	NM_214353	AGCACTGGGGAGAAAGGATT	AAAACTGGGAACCGTTTGTG
TBP	DQ845178.1	AACAGTTCAGTAGTTATGAGCCAGA	AGATGTTCTCAAACGCTTCG
CCL2	NM_214214.1	CCTCATCCTCCAGCATGAAGGTCTCTGC	GGTGGAGTCAGGCTTCAAGGCTTCGG
CD163	NM_213976	GATGTTCTGCCCATTTAAGTTCCT	TGACCAAGCAGGCACTGAGA
CIRBP	NM_001246197.1	GCATCAGACGAGGGCAAAC	CCCTGTCCTTCACTACTACCAC
FABP1	NM_001004046.1	ATCGTGCAGAATGGGAAGCA	ACTGAACCACTGTCTTGACC
GBP1	NM_001128473.1	AGAGCAAGAGAGGACGATGG	AAAGCGCTCTTCTCCAGTTC
G6PC	EU295557.1	CGGCTTTCGGTGCTTGAA	CTGCACAGTCCAGAATCCCA
PPARGC1A	NM_213963.1	CGCAAGCAATTTTTCAAGTCTAAC	GGAAGCAGGATCAAAGTCATCTG
