# Supplementary material for: Comparing the intestinal transcriptome of Meishan and Large White piglets during late fetal development reveals genes involved in glucose and lipid metabolism and immunity as valuable clues of intestinal maturity
Source: BMC Genomics. 2017 Aug 22;18:647. doi: 10.1186/s12864-017-4001-2 (PMC5568345; doi:10.1186/s12864-017-4001-2)

Additional file 5: Figure S1. PCA analysis of the additive model, the gestational age model and the fetal genotype model. (a) For the gestational age model, the PC1 explained 30.0% of the total variance and segregated data from fetuses according to their age. (b) For the genotype model, the data from purebred LW and MS fetuses were clearly separated along the PC1 (24.3%) while the data from crossbred fetuses segregated on the PC3 (10.5%). (c) The gestational age segregation was also clear cut in PC1 (24.4%) of the additive model, while the purebred fetuses segregated on PC2 (16.2%).

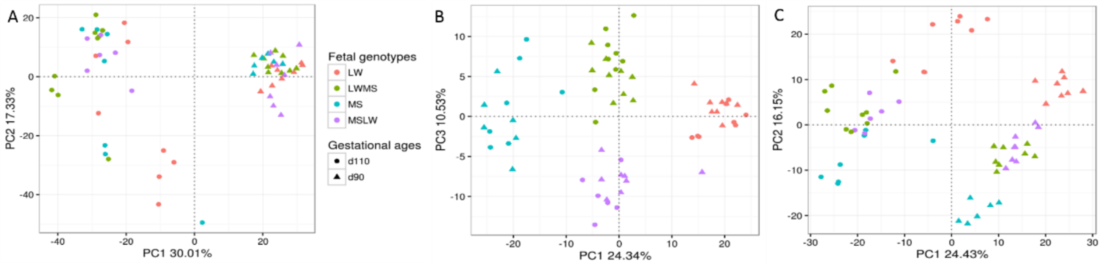

Supplement: Supplementary file 5 — PCA analysis of the additive model, the gestational age model and the fetal genotype model. (a) For the gestational age model, the PC1 explained 30.0% of the total variance and segregated data from fetuses according to their age. (b) For the genotype model, the data from purebred LW and MS fetuses were clearly separated along the PC1 (24.3%) while the data from crossbred fetuses segregated on the PC3 (10.5%). (c) The gestational age segregation was also clear cut in PC1 (24.4%) of the additive model, while the purebred fetuses segregated on PC2 (16.2%). (PDF 153 kb) [file 12864_2017_4001_MOESM5_ESM.pdf]
