# Supplementary material for: Comparing the intestinal transcriptome of Meishan and Large White piglets during late fetal development reveals genes involved in glucose and lipid metabolism and immunity as valuable clues of intestinal maturity
Source: BMC Genomics. 2017 Aug 22;18:647. doi: 10.1186/s12864-017-4001-2 (PMC5568345; doi:10.1186/s12864-017-4001-2)
Supplement: Supplementary file 8 — Box-plot representation of NR3C1 and TGFB2 expression in fetuses with different genotypes at 90 (d90) and 110 day (d110) of gestation. (PDF 120 kb) [file 12864_2017_4001_MOESM8_ESM.pdf]

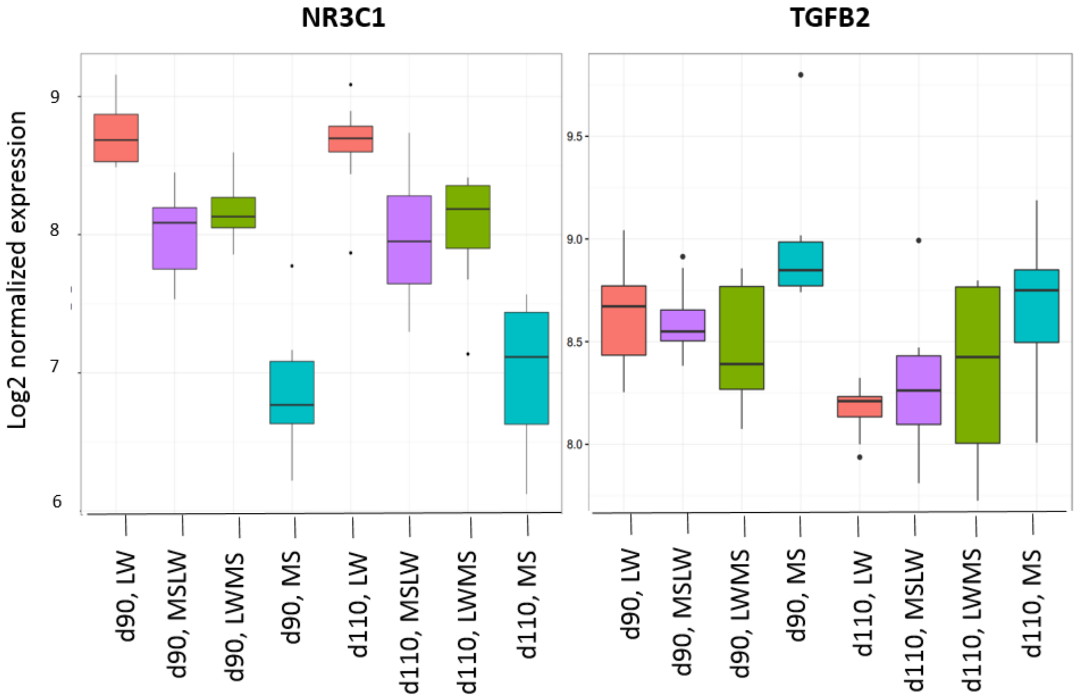

Additional file 8: Figure S2. Box-plot representation of NR3C1 and TGFB2 expression in fetuses with different genotypes at 90 (d90) and 110 day (d110) of gestation. LW, purebred fetuses from Large White sows; MSLW, crossbred fetuses from Large White sows; LWMS, crossbred fetuses from Meishan sows; MS, purebred fetuses from Meishan sows.
