# Supplementary figures and images for: Comparing the intestinal transcriptome of Meishan and Large White piglets during late fetal development reveals genes involved in glucose and lipid metabolism and immunity as valuable clues of intestinal maturity
Source: BMC Genomics. 2017 Aug 22;18:647. doi: 10.1186/s12864-017-4001-2 (PMC5568345; doi:10.1186/s12864-017-4001-2)

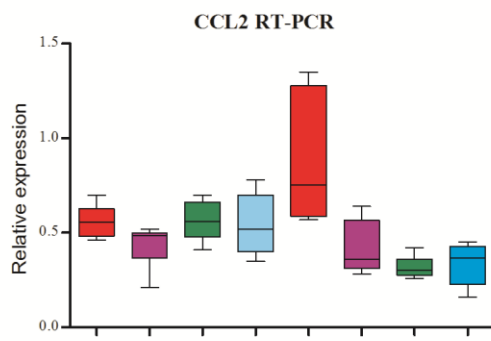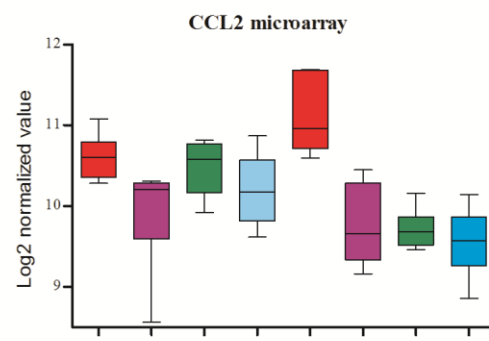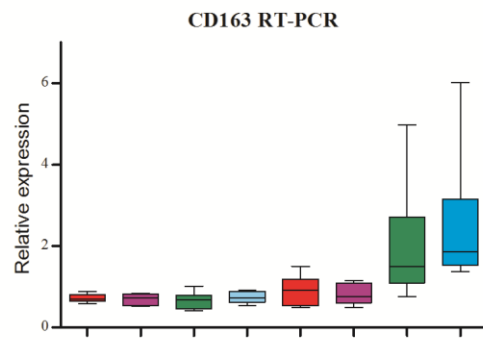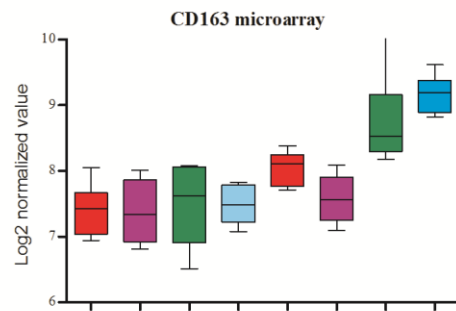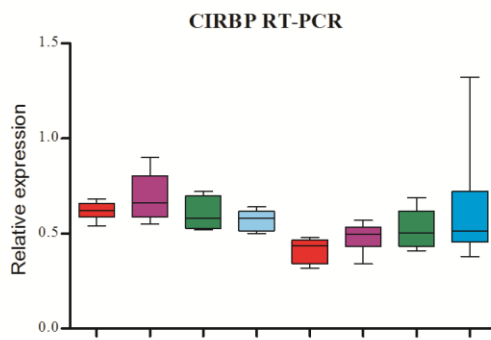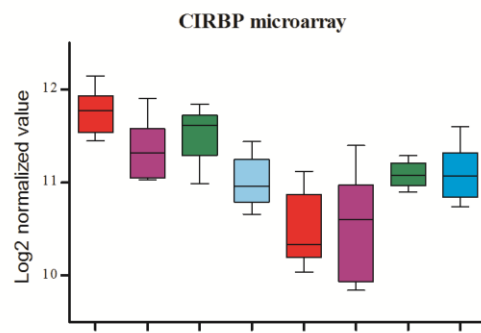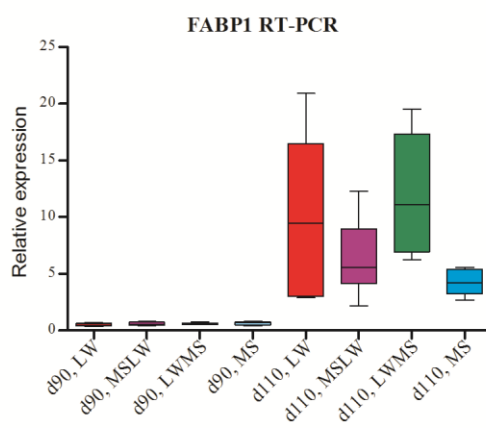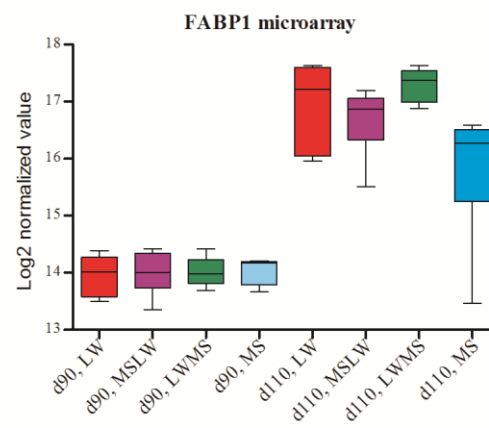

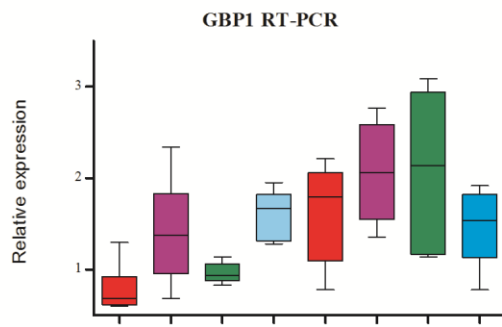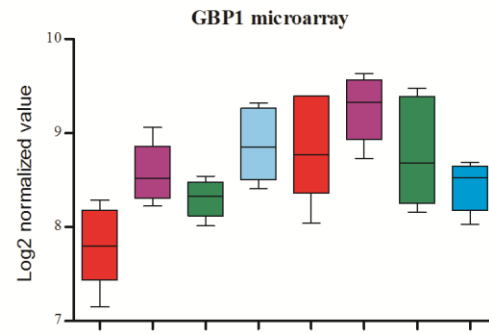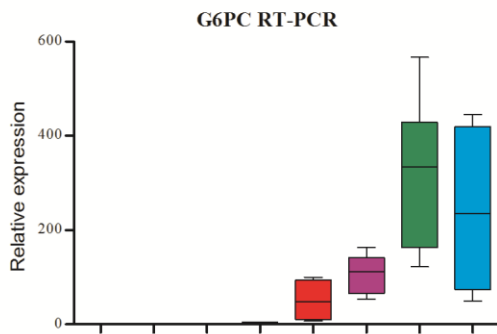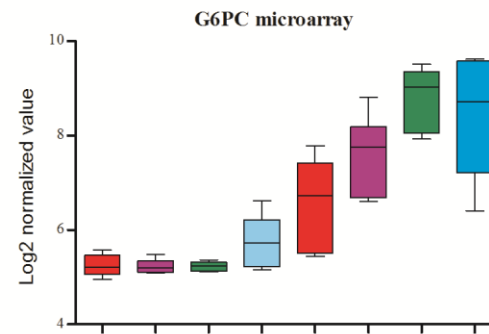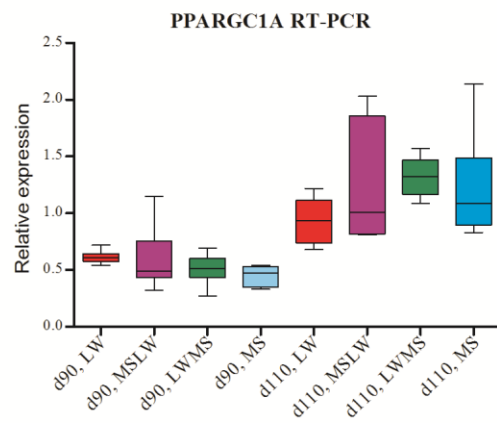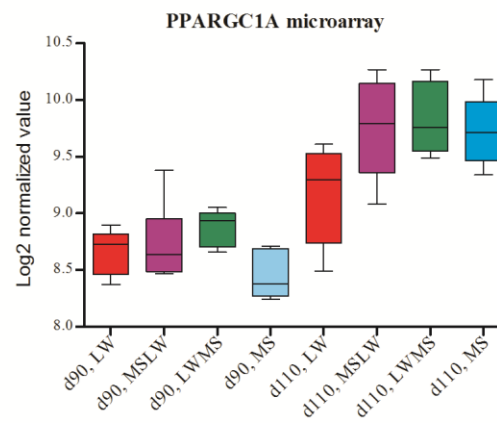

Supplement: Supplementary file 10 — Box-plot representation of the seven tested genes in qPCR compared to their microarray expression. (PDF 359 kb) [file 12864_2017_4001_MOESM10_ESM.pdf]
